# Can ACE2 expression explain SARS‐CoV‐2 infection of the respiratory epithelia in COVID‐19?

**DOI:** 10.15252/msb.20209841

**Published:** 2020-07-26

**Authors:** Martijn C Nawijn, Wim Timens

**Affiliations:** ^1^ Department of Pathology and Medical Biology University Medical Center Groningen GRIAC Research Institute University of Groningen Groningen The Netherlands

**Keywords:** Methods & Resources, Microbiology, Virology & Host Pathogen Interaction

## Abstract

Infection with severe acute respiratory syndrome coronavirus‐2 (SARS‐CoV‐2) leads to coronavirus disease 2019 (COVID‐19), which poses an unprecedented worldwide health crisis, and has been declared a pandemic by the World Health Organization (WHO) on March 11, 2020. The angiotensin converting enzyme 2 (ACE2) has been suggested to be the key protein used by SARS‐CoV‐2 for host cell entry. In their recent work, Lindskog and colleagues (Hikmet *et al*, 2020) report that ACE2 is expressed at very low protein levels—if at all—in respiratory epithelial cells. Severe COVID‐19, however, is characterized by acute respiratory distress syndrome and extensive damage to the alveoli in the lung parenchyma. Then, what is the role of the airway epithelium in the early stages of COVID‐19, and which cells need to be studied to characterize the biological mechanisms responsible for the progression to severe disease after initial infection by the novel coronavirus?

## Graphical abstract

The Angiotensin I converting enzyme 2 (ACE2) has been suggested as the key protein used by SARS‐CoV‐2 for host cell entry. In their recent study, Lindskog and colleagues (Hikmet *et al*, 2020) report very low ‐if any‐ ACE2 protein expression in respiratory epithelial cells.

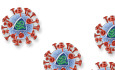

SARS‐CoV‐2 uses angiotensin‐converting enzyme 2 (ACE2) as receptor for docking and cell entry via the spike (S) glycoprotein. To elucidate the pathogenesis of COVID‐19, it is of critical importance to identify the cells infected by SARS‐CoV‐2, as well as the mechanisms of viral entry, replication, and the ensuing cellular damage leading to the induction of innate and adaptive antiviral responses. Several recent studies report on the expression of *ACE2* mRNA in tissues or individual cells, identifying the upper airway epithelium as the most likely site of entry for SARS‐CoV‐2 (Lukassen *et al*, 20202020; Sungnak *et al*, 20202020). In their recent study, Hikmet *et al* (20202020) present a much‐needed updated systematic evaluation of ACE2 expression in a large range of tissues, at the protein as well as the RNA level, confirming the expression in a number of epithelial barrier tissues. Importantly, the authors show that ACE2 expression in the respiratory tract is very limited compared to other barrier tissues, and reproducible staining is observed in nasal epithelial cells with only one of the two antibodies used in this study, prompting the authors to claim that none or only very low levels of ACE2 protein is present in the normal respiratory system.

Identification of the entry site of SARS‐CoV‐2 in COVID‐19 patients will facilitate focused research efforts to test whether and how the early response to viral infection governs progression to severe and fatal disease. Such insight into the pathogenesis of early stages of COVID‐19 might aid the identification of biomarkers for those patients most likely to progress to severe disease, and guide design of therapeutic interventions aiming at preventing severe disease in its variations. Finally, it might offer opportunities to further improve personal protection measures for those (healthcare) professionals at risk of attracting the virus. Several reports have started to explore the response of airway epithelial cells to SARS‐CoV‐2 infection. For instance, recent data indicate that primary airway epithelial cells have a relatively modest type‐1 interferon response to SARS‐CoV‐2 infection (Blanco‐Melo *et al*, 20202020), and their interactions with the immune system correlate to disease severity (Chua *et al*, 20202020). However, do we need to re‐evaluate a role for the airway epithelium as primary site of infection with SARS‐CoV‐2 based on the findings reported by Hikmet *et al*?

Their study reports on RNA and protein expression of ACE2 in a range of human tissues based on the Human Protein Atlas (HPA) resource. The tissue‐level RNA expression data align well with the single‐cell RNA sequencing (scRNA‐Seq) datasets re‐analyzed by the authors, although the nasal epithelium and the conjunctiva of the eye are lacking from the HPA dataset while showing relatively high *ACE2* expression in the single‐cell analyses (Sungnak *et al*, 20202020). The protein expression data for ACE2, however, represent the most relevant part of this study, with contrasting data in literature for ACE2 expression in the respiratory system (Hamming *et al*, 20042004; Jia *et al*, 20052005). The authors convincingly show expression in a range of tissues including duodenum, small and large intestine, the proximal tubule cells of the kidney, glandular cells in the gallbladder, and cardiomyocytes, as well as the conjunctiva of the eye. In the normal respiratory system, however, the authors observe positive staining only with one of two antibodies used, in ciliated cells in the nasal cavity (in all subjects tested), in the bronchus (in a subset of the subjects tested), and in a subset of alveolar type‐2 epithelial cells, and conclude that ACE2 protein expression in lung is very limited at best.

The baseline for ACE2 protein expression in normal tissue as reported by Hikmet *et al* is important for unraveling the pathogenesis of COVID‐19. Is the reported low level of ACE2 protein expression sufficient to support a role for the (upper) airway epithelium, in combination with the conjunctiva of the eye, as a first site of infection by SARS‐CoV‐2 as a prelude to developing COVID‐19? The nasal epithelial staining with the more sensitive antibody is reproducible, and the staining pattern matches that observed in the other epithelial barrier tissues. For the lower airways, however, staining with this antibody is not reproducible between donors. The antibody used in the earlier studies reporting positive staining in lower airways (Hamming *et al*, 20042004) was raised against a peptide sequence partly overlapping with the peptide used to generate the antibodies Hikmet *et al* used. Differences in recognition (and consequently in level of immunohistological expression) can also be due to differences in folding of the protein and thus differences in epitope expression. Moreover, *ACE2* has been shown to be an interferon‐induced gene (Ziegler *et al*, 20202020), and *ACE2* levels in airway epithelial cells dramatically increase during SARS‐CoV‐2 infection (Chua *et al*, 20202020). The presence of viral RNA has been observed in ciliated but not in secretory epithelial cells in the airways of COVID‐19 patients (Hou *et al*, 20202020), which is in line with the ACE2 staining pattern reported in this manuscript (Hikmet *et al*, 20202020), indicating that ACE2 expression might be sufficient to allow infection of the ciliated airway epithelial cells by SARS‐CoV‐2. In such a scenario (Fig 11), low‐level protein expression in upper airway epithelial cells facilitates infection of ciliated cells, followed by a rapid interferon‐induced increase of ACE2 expression in lower airways and lung parenchyma that then sets the stage for viral spread across the respiratory mucosa and which could contribute to severe COVID‐19.

**Figure 1:**
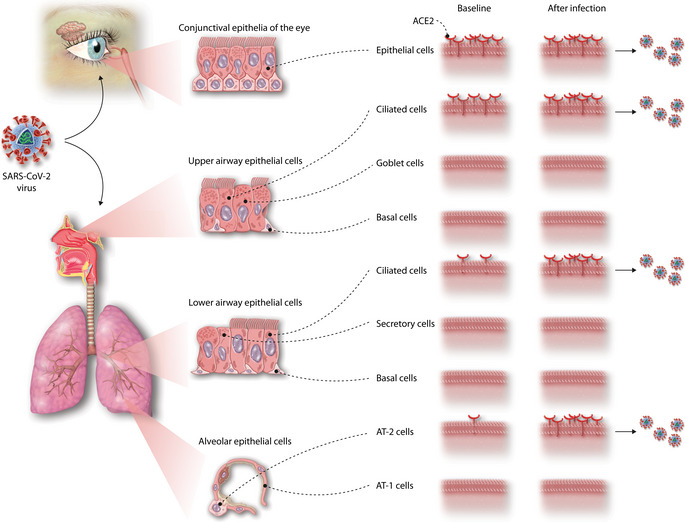
Proposed events during SARS‐CoV‐2 infection of the respiratory tract (A) At baseline, ACE2 expression is present in the conjunctival epithelia of the eye and at low levels in the ciliated epithelial cells in the upper airways, allowing SARS‐CoV‐2 infection at these initial sites. (B) The antiviral response induced upon SARS‐CoV‐2 infection induces a marked upregulation of ACE2 expression, allowing the SARS‐CoV‐2 to spread across the respiratory mucosa and into the parenchyma of the lung, where it can infect type‐2 alveolar epithelial cells.

In addition to baseline expression and changes thereof induced by SARS‐CoV‐2 infection, the effects of external factors and pre‐existing lung disease on ACE2 expression need to be considered. For instance, smoking has been shown to increase *ACE2* expression in the respiratory tract (Smith *et al*, 20202020). Expression of ACE2 in patients with pre‐existing lung disease also needs to be systematically evaluated, as very often these disorders are accompanied by significant increases in alveolar type‐2 epithelial cells, that are in the center of the main COVID‐19 lung pathology. Therefore, further studies are urgently needed on the role of respiratory epithelial cells during infection with SARS‐CoV‐2 and the early stages of COVID‐19, in particular in risk groups and patients with pre‐existing disease, to identify the complex mechanisms that lead to the exaggerated immune response seen in severe disease.
